# Cercariae of a Bird Schistosome Follow a Similar Emergence Pattern under Different Subarctic Conditions: First Experimental Study

**DOI:** 10.3390/pathogens11060647

**Published:** 2022-06-03

**Authors:** Miroslava Soldánová, Ana Born-Torrijos, Roar Kristoffersen, Rune Knudsen, Per-Arne Amundsen, Tomáš Scholz

**Affiliations:** 1 Institute of Parasitology, Biology Centre, Czech Academy of Sciences, 370 05 České Budějovice, Czech Republic; borntorrijos.ana@gmail.com (A.B.-T.); tscholz@paru.cas.cz (T.S.); 2 Department of Arctic and Marine Biology, Faculty of Biosciences, Fisheries and Economics, UiT The Arctic University of Norway, N9037 Tromsø, Norway; roar.kristoffersen@uit.no (R.K.); rune.knudsen@uit.no (R.K.); per-arne.amundsen@uit.no (P.-A.A.); 3 Department of Parasitology, Faculty of Science, University of South Bohemia in České Budějovice, 370 05 České Budějovice, Czech Republic

**Keywords:** trematodes, cercariae, bird schistosome, *Trichobilharzia*, emergence, light, temperature

## Abstract

The emergence of cercariae from infected mollusks is considered one of the most important adaptive strategies for maintaining the trematode life cycle. Short transmission opportunities of cercariae are often compensated by periodic daily rhythms in the cercarial release. However, there are virtually no data on the cercarial emergence of bird schistosomes from freshwater ecosystems in northern latitudes. We investigated the daily cercarial emergence rhythms of the bird schistosome *Trichobilharzia* sp. “peregra” from the snail host *Radix balthica* in a subarctic lake under both natural and laboratory seasonal conditions. We demonstrated a circadian rhythm with the highest emergence during the morning hours, being seasonally independent of the photo- and thermo-period regimes of subarctic summer and autumn, as well as relatively high production of cercariae at low temperatures typical of northern environments. These patterns were consistent under both field and laboratory conditions. While light intensity triggered and prolonged cercarial emergence, the temperature had little effect on cercarial rhythms but regulated seasonal output rates. This suggests an adaptive strategy of bird schistosomes to compensate for the narrow transmission window. Our results fill a gap in our knowledge of the transmission dynamics and success of bird schistosomes under high latitude conditions that may serve as a basis for elucidating future potential risks and implementing control measures related to the spread of cercarial dermatitis due to global warming.

## 1. Introduction

Schistosomes are digenetic trematodes that cause human schistosomiasis (species of *Schistosoma*) in the tropics and subtropics [1,2] and cercarial dermatitis worldwide, with species of *Trichobilharzia* being the most common causative agents of this water-borne eruptive skin disease in temperate zones [3,4,5]. Schistosomes have two-host life cycles, with mollusks serving as intermediate hosts in which the parasite reproduces through sporocysts (asexual reproduction or clonal multiplication [6,7]) to cercariae that emerge into the aquatic environment and infect mammals or birds as definitive hosts [4,5]. Snail–trematode interaction is a highly specific phenomenon, playing a crucial role in the successful parasite population build-up and host-to-host parasite transmission [8]. Schistosomes exhibit a complex range of physiological and behavioral adaptations that facilitate their dispersal to encounter and invade hosts, either through parasite-induced modifications in snails (e.g., [9,10,11,12]) or strategies directly used by their free-living stages, including cercariae (e.g., [13,14,15,16]). The emergence “strategy” of cercariae from infected mollusks is considered one of the most important adaptive strategies for maintaining the trematode life cycle [8,17].

The transmission opportunities of non-feeding and short-lived cercariae (usually a few days [18]) are often compensated by the timing of cercarial shedding with the most likely encounter with subsequent hosts [8,17,19]. Such synchronization allows cercariae to concentrate at high densities in host microhabitats at certain times of the day, thus enhancing the chance of successful transmission. Cercariae of most schistosome species emerge in a diurnally periodic manner with the highest numbers occurring during daylight and peaking once every 24 h (i.e., circadian rhythms) [20]. While a high diversity of chronobiological patterns is well documented for schistosomes infecting humans and other mammals (see review [20] and [21,22,23]), cercariae of bird schistosomes generally exhibit similar diurnal rhythms with a single peak occurring during the first period of the day [24,25,26,27,28,29,30,31,32,33,34], although a different periodicity with double peaks [35] or later midday/afternoon emergence was also reported [36,37]. Nevertheless, most information is limited to the most common and species-rich genus *Trichobilharzia* [3,4,5], particularly the host–parasite system *Lymnaea stagnalis*–*Trichobilharzia szidati* [29,30,31,33], where the morning emergence peak was attributed to the overlapping diurnal occurrence and high activity of both snail [27] and waterfowl hosts [31].

In addition to light exposure, the cercarial release from infected snails is strongly regulated by temperature, both with respect to emergence rhythms and increased cercarial output at elevated temperatures (e.g., [38,39,40,41]). Schistosome cercariae also show variations in daily emergence in response to temperature changes [20,33], often reflecting the seasonal climatic conditions of a particular geographical area [31,42,43,44].

Subarctic and Arctic regions represent highly challenging environments for parasite population dynamics because of a narrow transmission window [45]. The unique climatic conditions with generally low temperatures and strong seasonality (i.e., continuous light or dark days during short summer and long winter periods, respectively) have repeatedly been shown to affect various aspects of cercarial biology and ecology in marine ecosystems (reviewed in [46]). Yet, the cercarial emergence rhythms or the total daily production in freshwater habitats were poorly studied [47,48], and virtually no data are available for bird schistosomes. This contrasts with the amount of research on bird schistosome diversity and population ecology, and the incidence of cercarial dermatitis in northern areas [44,49,50,51,52,53,54,55,56,57,58]. Identifying adaptive behavioral strategies of trematodes in cold environments is important to understanding local parasite transmission dynamics, especially when facing the global threat of climate warming and the associated spread of parasitic diseases that are predicted to be most pronounced in high latitude environments [59,60,61,62].

In the present study, we investigated the daily cercarial emergence rhythm of a bird schistosome from an infected snail host in a subarctic lake. The study was conducted under various light and temperature conditions in both field and laboratory experiments, following the photo- and thermo-period regimes of subarctic summer and autumn. The main focus was on determining emergence peaks during the diel period, but given the lack of data, total output rates (i.e., the total number of emerged cercariae during 24 h) were also assessed. Based on the literature records, the effect of light is expected to be reflected in diurnal emergence, with chronobiological circadian patterns peaking once in the morning hours after the onset of illumination, regardless of the season. Temperature changes are expected to regulate seasonal cercarial output rates toward higher emergence in the summer. In contrast, we expect an overall mild temperature effect on cercarial diel rhythms due to small daily fluctuations in water temperature within each season in the subarctic lacustrine environment.

## 2. Results

Out of 3058 *Radix balthica* (Linnaeus, 1758) (Gastropoda: Lymnaeidae) examined between 2016–2018, 99 snails (3.2%) were infected with *Trichobilharzia* sp. “peregra” (sum of patent infections—revealed from cercarial release, and prepatent infections detected after snail dissections—from the presence of sporocysts and immature cercariae), of which 65 snails released cercariae, although none in October 2016 and 2017 (Table 1). Almost all experimental snails survived throughout the field experiments in all months (except for August 2017), but there was a decreased survival under laboratory conditions (Table 1, Appendix A Table A1). Although we repeatedly confirmed that all *Trichobilharzia*-infected snails shed cercariae prior to the initial experiments in the field, some individuals showed zero or very low emergence during the experiments (August 2017 and especially October 2018; Appendix A Table A1). Seven snails harbored double infections with an additional trematode species present, most of which were detected after snail dissections with immature cercariae in sporocysts, especially in October 2018 (Appendix A Table A2). The only simultaneous patent infection was observed in August 2016, when mature cercariae of *Plagiorchis* sp. emerged immediately at the first counting in the field and remained patent until the end of laboratory experiments three days later (snail no. 7 T-A16, Appendix A Table A2). The total number of *Plagiorchis* cercariae released per day was generally higher compared to bird schistosome cercariae (day 1-field, 216 vs. 96; day 2-field, 60 vs. 8; day 3-laboratory, 930 vs. 39, respectively; see also numbers of *Trichobilharzia* sp. “peregra” in Appendix A Table A1).

### 2.1. Daily Patterns in Cercarial Emergence

**Figure 1:**
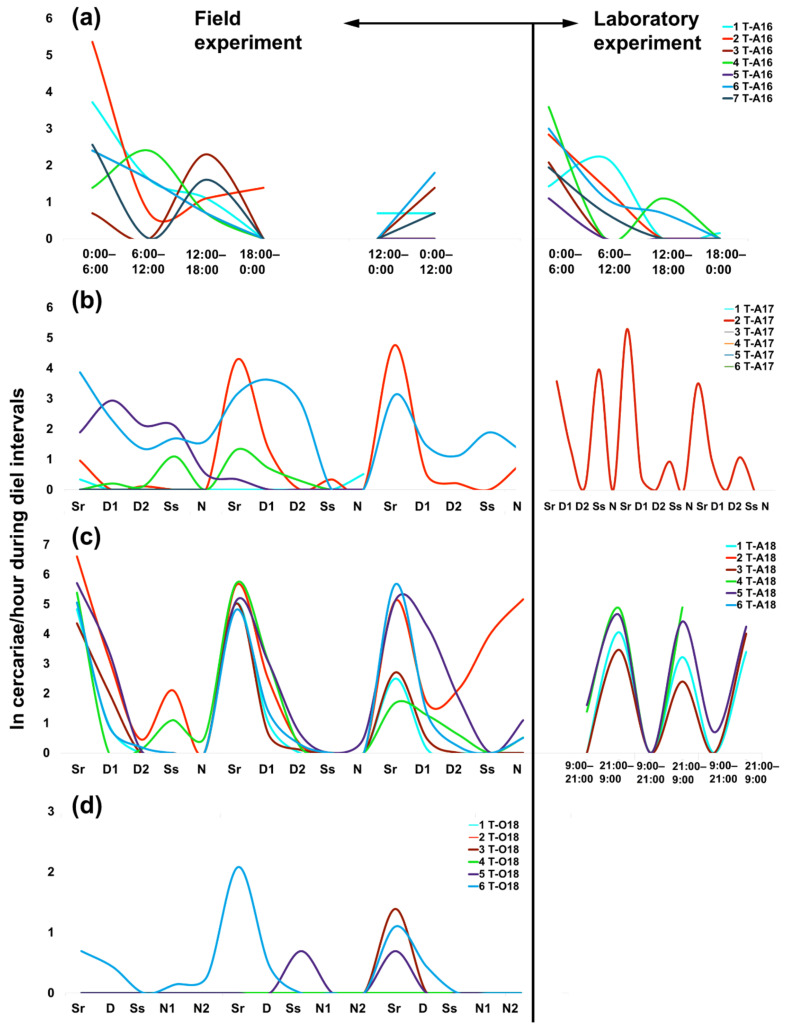
Daily patterns in cercarial emergence (recalculated to 1 hour) of *Trichobilharzia* sp. “peregra” from individuals of *Radix balthica* snails (marked with different line colors) during diel intervals (Sr, sunrise; D, day; Ss, sunset; N, night) under field and laboratory conditions in (**a**) August 2016, (**b**) August 2017, (**c**) August 2018, and (**d**) October 2018. Cercarial emergence during the longest parts of the natural photoperiod in summer (day interval in August) and autumn (night interval in October) are divided into half and termed as D1, D2 and N1, N2. Note the different number of experimental days in August 2016 (see also Table 1). The emergence data from October 2018 are not shown due to a single snail producing cercariae in similar numbers during a single day (see Appendix A Table A1). “ln” refers to ln-transformed number of emerged cercariae per hour.

**Figure A1:**
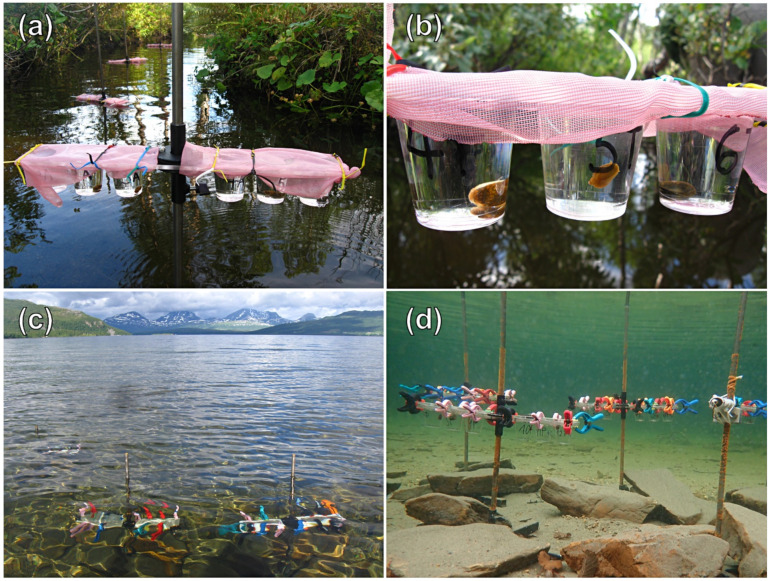
*In situ* emergence of cercariae of *Trichobilharzia* sp. “peregra” from naturally infected snails *Radix balthica*. (**a**,**b**) Experimental design conducted in a side stream flowing into Lake Takvatn during the preliminary testing of constructions in 2016. (**c**,**d**) Experimental design conducted directly in the lake between 2017–2018.

Cercarial emergence was diurnal as the maximum number emerged during daylight, regardless of the different experimental designs (Appendix A, Figure A1) or different natural daylight conditions with 20 h in August and 10 h in October (Table 2). The diurnal pattern was especially evident in experiments following specific diel intervals with the length of daylight starting from sunrise to the beginning of sunset (Table 2, see Appendix A Table A3 for light intensity). Recalculation of the data to 1 h emergence suggested the highest shedding during sunrise in all years and seasons, indicating periodic output with circadian rhythms, i.e., a single peak within 24 h (Figure 1). To some extent, this also applies to the 6 and 12 h intervals used in the field and laboratory experiments in August 2016. Most cercariae emerged between 0:00–6:00, which covers mainly nighttime and sunrise, or between 0:00–12:00 covering night, sunrise, and the first half of the day (Figure 1a; see also “additional data” in Appendix A Table A3). Although different diel intervals were used in August 2016, the emergence peaks can be clearly inferred from the literature records and our emergence data obtained in subsequent years when cercarial shedding in the field was monitored individually over five diel intervals, supporting present and previous literature findings on the diurnal rather than nocturnal emergence of *Trichobilharzia* sp. “peregra” cercariae. Twelve-hour intervals in the laboratory experiments repeatedly showed a high emergence between 21:00–9:00 in August 2018 (Figure 1c, Appendix A Table A1), but this pattern was not evident in October 2018 when only a single snail produced cercariae in similar numbers during a single day (Appendix A Table A1).

**Figure 2:**
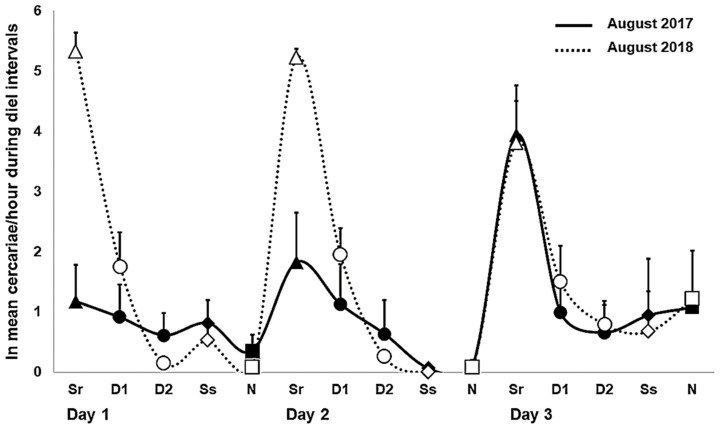
Daily patterns in cercarial emergence (mean ± SE, recalculated to 1 h) of *Trichobilharzia* sp. “peregra” from *Radix balthica* snails during diel intervals (Sr, sunrise in triangles; D, day in circles; Ss, sunset in diamonds; N, night in squares) under natural photoperiod in field experiments in August 2017 and 2018. Day interval is divided into half and termed as D1 and D2. Note that the mean value of cercarial emergence from two snails is given for the third day of the experiment in August 2017 (see Appendix A Table A1). “ln” refers to ln-transformed number of emerged cercariae per hour.

The general trend of the highest cercarial emergence during sunrise also showed some variance among experimental setups and snail individuals (Figure 1). Additionally, increased emergence was detected in August 2016 between 12:00–18:00 (both in the field and laboratory) (Figure 1a), covering mainly the second half of the day (Appendix A Table A3), whereas the next highest values were observed during the first half of the day in the subsequent years, including three snails that released cercariae in the field setup in October (Figure 1b–d, Appendix A Table A1). Snails showed the most inconsistent shedding on the first day of the field experiment in August 2017 (Figure 1b), likely resulting in reasonably comparable numbers of cercariae between sunrise and both day intervals (Figure 2). During the following two days, emergence was more homogeneous in terms of increased production at sunrise, followed by the first half of the day (Figure 1b and Figure 2). This was mainly due to two snails, from one of which cercariae maintained the same periodic emergence pattern also in the laboratory (snail no. 2 T-A17, Figure 1b). The most consistent emergence rhythms among snail individuals were observed in August 2018 (Figure 1c), with a recurrent high production of cercariae at the same time of the day across all experimental days (Figure 2).

Daily emergence of cercariae did not correlate with fluctuations in water temperature or light intensity at specific diel intervals (Table 3).

### 2.2. Daily Output in Cercarial Emergence

The daily output rates of cercariae in the field differed between years and seasons, with a higher emergence in August than in October, as well as in August 2018 compared to August 2016 and 2017 (Table 1). Cercarial production between laboratory and field experiments seemed variable, but this may be biased by different experimental conditions and the number of snail replicates. Only one snail surviving both experiments in August 2017 and three snails in August 2018 could be compared, in both cases showing higher production in the laboratory than in the field (no. 2 T-A17, 298 vs. 169 cercariae snail/day; nos. 1, 3, 5 T-A18, mean of 619 vs. 459 cercariae snail/day; Appendix A Table A1). Similarly, one snail in October 2018 shed more cercariae in the laboratory (no. 6 T-O18, 21 vs. 7 cercariae on the third experimental day; Appendix A Table A1). The total cercarial production varied among snail individuals, with some hosts showing higher shedding than others (Figure 1, Appendix A Table A1). However, this was not related to snail size (Table 3), although shell lengths varied considerably within each month in a given year (Appendix A, Table A2). A positive correlation between snail size and cercarial output was found only in October 2018 (Table 3), being due to the highest emergence occurring from the single largest snail individual (Appendix A Table A1 and Table A2). Snails did not differ significantly in the mean shell length among seasons and years (one-way analysis of variance, ANOVA; F_3,21_ = 0.838, *p* > 0.05), indicating comparable snail sizes and thus a negligible effect of the snail size on the seasonal/annual variation in cercarial output rates.

## 3. Discussion

This study is the first to investigate the cercarial emergence of the bird schistosome *Trichobilharzia* sp. “peregra” from their *Radix balthica* snail hosts under different seasonal light and temperature conditions in a subarctic lacustrine environment. Current knowledge on the cercarial emergence patterns and rates from molluscan hosts in the Arctic and subarctic is still scarce and almost exclusively includes marine trematodes ([46]; but see [47,48] for examples of freshwater trematodes). Interestingly, despite their global distribution and importance in the epidemiology of human cercarial dermatitis [4,5,63], there are no data on bird schistosomes, which hinders our understanding of their transmission dynamics and success in completing the life cycle at high latitudes. Over three years of field and laboratory experiments, we were able to fill this knowledge gap by repeatedly demonstrating a circadian rhythm in emergence (one peak within 24 h) and relatively high production of cercariae at low temperatures typical of northern environments. This suggests an adaptive strategy of bird schistosomes to compensate for the narrow transmission window that allows them to maximize the probability of reaching a suitable host under the hostile subarctic climate.

In accordance with our first expectation based on literature records on the cercarial emergence of bird schistosomes, the cercariae of *Trichobilharzia* sp. “peregra” seem to exhibit a diurnal rhythm, with peak emergence occurring immediately after the onset of illumination at sunrise. This was most evident in field experiments under a natural photoperiod, but also when simulating field conditions in the laboratory, despite the low number of snail replicates (a single snail in August 2017) or 6 and 12 h diel intervals (August 2016 and 2018). Patterns in cercarial emergence are often trematode-specific [8,64,65] and this is particularly evident in mammalian schistosomes through a wide variety of emergence peaks along the daily photocycle [8,20,21,22,23]. Bird schistosomes appear to differ in this regard, with most species showing a uniform chronobiological pattern with the highest shedding in the morning hours. Our results are broadly consistent with studies examining various species of *Trichobilharzia* and snails from different latitudes in Europe and North America between 44°–56°N, where most research efforts have focused [24,25,26,27,29,30,31]. Combined with our data from subarctic Lake Takvatn (69°N), this indicates that the periodic cercarial emergence of *Trichobilharzia* species is probably independent of the snail host species and parasite geographical distribution, and thus of the wide range of seasonal temperature and light conditions under which they live.

In addition, cercarial rhythms of *Trichobilharzia* sp. “peregra” were consistent in both summer and autumn, indicating a stable seasonal pattern in the occurrence of emergence peaks, similarly to other trematodes and schistosomes [31,42,65]. Although we rely on only three snails that released cercariae in October 2018, we believe that a similar rhythm of shedding would also be observed if larger numbers of snail replicates were available. The only prepatent and patent infections of *Trichobilharzia* sp. “peregra” found in the autumn were likely due to the exceptionally warm summer in August 2018, which resulted in about 2 °C warmer water in the lake, accelerating trematode development and thus cercarial release in the autumn [39,40,48,64,66]. This provided unique emergence data that are otherwise difficult to obtain, as the prevalence of bird schistosomes in snails is generally low in both temperate (typically between 0.05 and 5%, [4,5]) and subarctic latitudes (<2%, present study, [44,49,52]) and decreases toward autumn.

Light and temperature are the most important and often interacting factors affecting the emergence of cercariae from snail hosts (e.g., [8,17,38,39]). Our study revealed that the emergence of *Trichobilharzia* sp. “peregra” was also strongly light- and temperature-dependent, but with different effects on daily emergence rates and rhythms. First, the diurnal shedding was prolonged during polar days in summer with 20 h continuous daylight, consistent with the extensive evidence from marine trematodes in Arctic areas [48,64] (reviewed in [46]) and the fact that light is the major regulator of the emergence of schistosomes [20,31], but see examples of nocturnal chronotypes of *Schistosoma* spp. [20,21,22,43]. This suggests that the capacity for higher cercarial output is greater during the key seasonal transmission window than in temperate latitudes, which is considered an important adaptive mechanism favoring transmission in cold-water environments [46]. Second, daily rhythms in cercarial emergence at high latitudes were also attributed to the influence of photoperiod, especially in species with ocellate cercariae whose maximum emergence coincides with the daily maximum light intensity [48,64]. Bird schistosomes are particularly sensitive to light changes [5,8,14,16] because their ocellate furcocercariae are equipped with two pigmented eye spots that facilitate the orientation of cercariae when searching for a definitive host. However, the daily rhythms of *Trichobilharzia* sp. “peregra” in our study were only light-regulated such that the release of almost all cercariae was triggered at sunrise, followed by a steady decline during the day and inhibition at dark. This is confirmed by the highest shedding in the morning when light intensity was still low (range of 11–236 Lx during different seasons and years). Nonetheless, light intensity appeared to influence individual emergence patterns from snail hosts, depending on weather changes. While heterogeneous emergence in summer 2017 was likely determined by variable conditions, alternating rainy, cloudy and sunny days, more stable rhythms were observed during sunny weather in summer 2018. Prokofiev et al. [48] suggested that the transparency of snail shells and associated light transmittance may contribute to species-specific emergence rhythms of ocellate cercariae. Although the strong photosensitivity of bird schistosomes is mainly reflected in the behavior of cercariae after their release from the snail, this could be a possible explanation for the annual and between-snail discrepancies in the periodic emergence observed in our study. In addition, cercarial rhythms may exhibit a certain degree of plasticity in response to the local biotope structure in which the emergence of cercariae occurs [35,67,68]. Accordingly, the two emergence peaks found in August 2016 may have resulted from different environmental conditions between the tree-shaded habitat of the side stream and the open space of Lake Takvatn. Evidence for the adaptive benefits of bird schistosomes in subarctic environmental conditions may represent a recently documented somatic dimorphism in cercariae of *Trichobilharzia* sp. “peregra”, with a large phenotype that may facilitate parasite transmission [58].

Consistent with our second expectation regarding the effect of temperature, the daily temperature fluctuations during the different diel intervals were too small to regulate the emergence patterns of *Trichobilharzia* sp. “peregra”. In freshwater ecosystems at northern latitudes, daily and seasonal variations in water temperature (in terms of differences between minimum and maximum values) are less pronounced (present study, [69]) and this is in sharp contrast to marine environments, where rhythms of cercarial emergence clearly correlate with the daily dynamics of seawater temperature, in addition to light intensity [46,48,64]. However, temperature regulated the overall seasonal and annual variations in *Trichobilharzia* sp. “peregra” cercarial output rates. As expected, the pronounced effect of temperature was evidenced by the high emergence in summer compared to the low shedding in autumn, and the 4- to 5-fold higher emergence in August 2018 compared to August of other years, which was due to the unusually warm temperatures in the lake reaching up to 17 °C. Since temperature usually leads to increased output rates (up to an optimal range [40]), our results confirm the commonly known phenomenon of the extreme sensitivity of trematodes to temperature changes [38,39,40,41,70], including schistosomes [20,33]. Such a remarkable rise in temperature could have important ecological and epidemiological implications, especially at high latitudes where the effects of climate change will be most pronounced. It was predicted that the spread of parasites, infections and disease incidence will increase substantially in a warming environment [59,60,61,62], as a result of extended summer periods and the transmission window for parasites [45]. For example, a 5-fold increase in cercarial emergence was predicted for *Trichobilharzia* sp. from *Radix peregra* with a 10 °C increase in temperature [39]. This suggests that higher cercarial emergence of bird schistosomes may be accompanied by a more frequent incidence of human cercarial dermatitis in the future, as it may occur beyond the main summer season. However, recent studies suggest that global warming may not be as pronounced in intensifying trematode transmission due to the thermostability of cercarial emergence over the normal temperature range to which the parasite is otherwise acclimated [40,46]. In addition, trematode cercariae, including the bird schistosome in northern areas [55,56], are frequently consumed by natural predators [71,72,73], which may thus benefit from the increased food supply while reducing cercarial populations.

Several factors other than light and temperature could have contributed to the variable output rates, either at the total (across all snails) or individual emergence level. For example, the increased cercarial output could be due to larger snails providing more space for parasite multiplication and subsequent cercarial production [65,74,75]. Although the infected snails in our study varied in size within each season of a given year, no significant effect on the cercarial output was detected (except in October, when a single larger snail shed more cercariae). In contrast, snails did not differ in the mean length among seasons and years, suggesting that temperature, rather than snail size, was the principal factor regulating the magnitude of the cercarial output of *Trichobilharzia* sp. “peregra”. In addition, multiple infections with trematodes in snail hosts usually lead to reduced cercarial production and eventually to the exclusion of the less competitive subordinate species [76,77,78]. In our study, all trematodes involved in co-infections were sporocyst-producing parasites with either strong (*Diplostomum*, *Plagiorchis*) or weak (*Apatemon*, *Cotylurus*) competitive ability towards the generally subordinate *Trichobilharzia* [78]. However, at the time of our study, the cercarial emergence of *Trichobilharzia* sp. “peregra” appeared to be unaffected, as the number of cercariae emerging from double-infected snails was in the range or even higher than that observed from single infections. Other factors such as multiple genotypes of conspecific trematodes, the condition of the snail host in relation to food availability, age and intensity of infection, or experimental conditions may also cause variation in the cercarial output [29,74,79,80,81]. In contrast to some previous findings of significantly higher output under artificial light conditions characterized by more intense illumination (e.g., [74]), cercarial emergence in our study was investigated in a specialized climatic chamber (laboratory treatments) simulating the natural photo- and thermo-period of seasonal climatic conditions in the subarctic. Although a slight increase could be detected (but the evidence was from a few snails), the emergence of cercariae of *Trichobilharzia* sp. “peregra” in the laboratory followed the same consistent pattern as in the field.

The cercariae emerge from the snails as a result of a stimuli-dependent process (temperature and/or light) that is also usually synchronized with the chronobiological behavior of the next host in the parasite’s life cycle when the chances of a mutual encounter are higher [8,17,19]. There is ample evidence of the adaptive abilities of mammalian schistosomes to optimize host-to-host transmission through the periodic release of cercariae [20,21,22,23], and such ecological traits of concentrating cercariae in host space were also suggested for bird schistosomes [31]. In Arctic seas, emergence rhythms were also found to coincide with the host behavior and related searching behavior of marine cercariae [64]. In Lake Takvatn, up to 21 species of aquatic birds were recorded over the years during the breeding season [82], of which four common duck species (*Anas penelope*, *Anas platyrhynchos*, *Aythya fuligula*, *Bucephala clangula*) are considered possible definitive hosts of *Trichobilharzia* sp. “peregra” [54]. The cercarial emergence was triggered by light, with the highest shedding occurring in the morning, when most of these hosts are also most active [83,84], indicating that the timing of cercarial shedding coincides with host availability also in cold subarctic regions. Together with the consistent emergence patterns of several bird schistosome species, this suggests that such adaptive behavior enhancing the probability of successful transmission may be a common feature of these parasites. However, further studies are needed to include different snail–bird schistosome systems from different geographical areas to investigate the possible variation in cercarial emergence rhythms resulting from adaptations to local environmental conditions, as demonstrated in mammalian schistosomes (e.g., [20,21,22,23]).

In summary, our study highlights an important feature for the successful transmission of bird schistosomes in subarctic environments, namely well-established adaptations to adverse climatic conditions. This was evident in prolonged emergence during summer, relatively high output rates in cold waters, and some degree of polymorphism in cercarial emergence in response to local environmental conditions. These observations complement our previous findings on the functional and ecological roles of bird schistosomes and other trematode taxa [55,56,57,58] in probably the best-studied lake ecosystem in northern Norway in terms of parasite and wildlife ecology and biology, and their interactions in food webs [85]. In addition, our study contributes to a better understanding of the transmission strategies of bird schistosomes at high latitudes, which is important to better recognize the mechanisms behind infection dynamics, circulation, and persistence of parasite populations. This can serve as a basis for elucidating future potential risks and implementing control measures related to the spread of cercarial dermatitis due to global warming.

## 4. Materials and Methods

### 4.1. Snail and Parasite Material

*Radix balthica* snails were collected by hand in the littoral zone (<1 m depth) of the subarctic freshwater Lake Takvatn in northern Norway (69°07′ N, 19°05′ E). Takvatn is an oligotrophic dimictic lake at an altitude of 214 m above sea level, with a surface area of 15 km^2^ and a maximum depth of ca. 80 m. The lake is usually ice-free for 5–6 months from mid-June to late November. *Radix balthica* is the dominant molluscan species and first intermediate host for the majority of larval trematodes in Takvatn (19 of 24 species/lineages), of which *Trichobilharzia franki* haplotype “peregra” *sensu* Jouet et al. (2010) (hereafter called *Trichobilharzia* sp. “peregra”; [86]) is the only bird schistosome of the genus *Trichobilharzia* [54,55,56,58].

Sampling and emergence experiments were conducted from 2016–2018 in August and October, representing two seasons (summer and autumn) with distinct water temperature and light conditions (usually 13 °C and 6 °C; light:dark photoperiod 20:4 h and 10:14 h, respectively). In total 3058 snails were collected and individually screened for trematode patent infections (cercarial release), followed by snail dissections for prepatent infections (intramolluscan stages, sporocysts and/or rediae, depending on the trematode species) when no cercarial shedding was observed for two days in order to determine the overall and *Trichobilharzia* sp. “peregra” prevalence in snail populations (Table 1). Snail processing and *in vivo* morphological identification of trematode larvae followed the methodology according to previous experience with larval trematode diversity in snails in Lake Takvatn [54,55,56,57]. The identification of trematodes involved in double infections (i.e., co-occurrence of two trematode species within a snail individual) was possible at the genus/species level due to the presence of almost mature cercariae. Experiments were performed in both months whenever a sufficient number of *Trichobilharzia*-infected snails were found (Table 1). Although there were more infected snails available in some seasons, a minimum of 6 snails infected with *Trichobilharzia* sp. “peregra” were selected each month to have a similar number of replicates to the first experiment in August 2016, resulting in a total of 25 experimental snails during the course of the study (Table 1). All experimental snails were measured for shell length and width using a digital calliper MarCal 16 EWR (accuracy 0.01 mm) (Mahr, Esslingen am Neckar, Germany) and fed lettuce (*Lactuca sativa*) until the start of the acclimatization process when snails were kept without food also during all field and laboratory experiments. None of the experimental snails harbored double infections prior to the start of experiments. Snails were dissected at the end or during experiments (due to spontaneous death) to confirm their infection status.

### 4.2. Experimental Setup and Monitoring of Cercarial Emergence

Cercarial emergence of *Trichobilharzia* sp. “peregra” was monitored in two types of experiments: (i) in the field under natural photo- and thermo-periods, and (ii) in the laboratory under simulation of field conditions. 

(i) In the field experiments, experimental snails were placed into 40 mL transparent plastic beakers filled with filtered lake water (using a 12 µm pore filter membrane, Whatman, Nuclepore Track-Etch Membrane, Sigma-Aldrich, Germany) and attached to an experimental metal device designed to immerse the beakers in the water (Appendix A Figure A1). At the same time, four beakers containing water without snails were used to control for possible contamination by cercariae from the outside water, but no cercariae were observed during the course of the experiments. After identifying all patent infections (approximately one day), infected snails were acclimatized to the ambient photo- and thermo-period of each season by maintaining beakers with snails *in situ* for two days prior to the start of the experiments. 

(ii) The laboratory experiments were conducted after a three-day acclimatization period of snails in a climatic chamber; a temperature- and irradiance-controlled room allowing an automatic adjustment to specific values according to daily and seasonal temperature and light fluctuations in the field. The lake water was tempered to the selected temperature before the laboratory experiments.

Temperature and light intensity parameters were measured every five minutes using data loggers (Onset HOBO UA-002-64 Pendant 64 K) during all field and laboratory experiments. In addition, weather condition was visually monitored in the field in terms of recording sunny, rainy and cloudy days. The meteorological conditions within daylight during the field experiments in August 2016 consisted of the first two days with alternating sunny and cloudy weather and the third being a sunny day. In August 2017, the first day was mostly cloudy, while it was sunny in the mornings with occasional overcast until noon, followed by cloudy and rainy afternoons with occasional sunshine during the remaining two days of experiments. In August 2018, all three days were sunny. In this year, an unusually warm summer was recorded throughout Europe with exceptionally high Arctic temperatures relative to the long-term average and the hottest summer ever recorded in Norway, with air temperatures even exceeding 30 °C, which is 10 to 15 °C above the normal range for July and August [87]. Typical subarctic autumn weather with cloudy and rainy days, sometimes with light snowfall, was observed in October 2018. 

Daily rhythms in cercarial emergence were determined by counting the number of released cercariae during specific time intervals over the diel periods (i.e., diel intervals), enabling us to identify emergence peaks. However, a different number of intervals per day was used in the field and laboratory experiments (Table 1, Appendix A Table A1), depending on the experimental setup, the tested questions and our parallel research activities in 2017 and 2018 [55,56,57]. In particular, the experimental setup in August 2016 differed on several points as it was a pilot study to test the suitability of using experimental metal constructions in the field, whether cercariae are released at low temperatures, and to determine the total daily production under the subarctic climate. First, the experimental constructions were originally designed for only a partial immersion of the beakers, which were covered with a breathable fabric to prevent the snails from escaping. However, this proved to be unstable in the lake due to possible strong wave activity. *In situ* emergence was therefore conducted in a side stream flowing into Lake Takvatn (69°05′186″ N, 19°08′160″ E, Appendix A Figure A1a,b). Second, the cercarial release was investigated over 1–2 days in 6 and 12 h diel intervals due to a limited number of persons counting cercariae (Table 1). Third, the laboratory experiment was conducted in the incubator with light conditions set to a 20:4 h light:dark photocycle (darkness from 22:00 to 2:00, see also “additional data” in Appendix A Table A3), but natural fluctuations in daylight illumination could not be simulated. Although the water temperature in the stream was much colder than in the lake (~8 °C vs. ~13 °C), the incubator was set to the usual lake temperature of 13 °C in summer/August (Table 1).

In subsequent years 2017 and 2018, the experimental constructions were improved to be fully submerged directly in the lake (at a depth of 15 cm and a distance of 3 m from shore), using a transparent lid covering the beakers with experimental snails (Appendix A Figure A1c,d). All field experiments in 2017 and 2018 lasted three consecutive days, during which cercariae were counted according to the natural daylight regime of sunrise, day, sunset and night (Table 1, Appendix A Table A1 and Table A3). To control for possible variations in cercarial emergence during the longest parts of the natural photoperiod in summer and autumn, day and night intervals (August and October, respectively) were divided into half, resulting in a total of five counts/five diel intervals during 24 h (Table 1, Appendix A Table A1). In the laboratory, only the experiments performed in August 2017 followed this design, whereas cercarial emergence monitored every 12 h in August and October 2018 was primarily designed to compare daily output rates with those under field conditions (Table 1, Appendix A Table A1). Temperature and light intensity in the climatic chamber were set up based on data recorded in the field by data loggers (Table 1, see also Appendix A Table A3).

Daily rhythms with emergence peaks were determined according to the protocol described in Vyhlídalová and Soldánová [65]. Briefly, after transferring the snails to new beakers at each counting interval, 1 mL from the homogenized 40 mL volume of water containing cercariae was taken ten times from each beaker to count cercariae under a stereomicroscope Zeiss Stemi DV4 (Carl Zeiss Microlmaging GmbH, Göttingen, Germany). The total production of cercariae was estimated for the 40 mL volume and calculated separately for each diel interval and individual snail replicates on each experimental day (Appendix A Table A1), as well as on average across snail replicates for each experimental day separately (Table 2). Raw data (observed number of emerged cercariae) obtained from both types of experiments and seasons/years were then converted to a 1 h emergence (i.e., the number of emerged cercariae was divided by the duration of each diel interval in minutes and the total resulting value was multiplied by 60) to identify possible circadian rhythms in the cercarial emergence (including data originating from 6 and 12 h intervals to standardize results). In addition, the total daily output rates (mean number of cercariae produced by a snail individual per 24 h, i.e., per snail/day), were determined by pooling observed number of cercariae across diel intervals, snails and days (Table 1).

### 4.3. Data Analysis

The use of different diel intervals (August 2016), high mortality rates of infected snails resulting in an incomparable number of replicates (August 2017), and the low numbers of emerged cercariae in general (October 2018) or during some intervals (August 2018) (Appendix A Table A1) make statistical analysis of daily patterns in cercarial emergence problematic. Similarly, it was not possible to statistically compare cercarial output rates between the field and laboratory experiments or among seasons and years. Given the main focus of our study, we therefore simply plotted the number of cercariae emerging daily from individual snails per 1 h to visually compare the consistency of circadian rhythms across seasons and years, i.e., general patterns in cercarial emergence. Emergence rhythms were then evaluated in relation to temperature and light conditions, as these factors mainly regulate the release of cercariae [e.g., 64,70], including schistosomes [20,31,33].

Daily output rates were tested in relation to snail size (shell length) because larger snails usually produce more cercariae [65,74,75]. Emergence data provided here are based on different sets of naturally *Trichobilharzia*-infected snails collected at different years and seasons, and a selection of snails with similar sizes was impossible due to low number of available replicates or low shedding (Table 1, Appendix A Table A1). Hence, the experimental snails used in each month varied in size (Appendix A Table A1) and there was also variability in the mean shell length (± SD) among seasons and years (August 2016, 13.90 ± 2.02 mm; August 2017, 12.47 ± 2.54 mm; August 2018, 11.73 ± 1.58 mm; and October 2018, 13.19 ± 3.86 mm). A Pearson correlation test was applied to the relationship between temperature or light intensity and the number of cercariae (pooled across all snails) corresponding to each diel interval, whereas the effect of snail size on the number of cercariae was analyzed using the observed cercarial output from each snail in each month. Differences in snail size (natural-log-transformed) among seasons and years were tested using analysis of variance (one-way ANOVA). All analyses were performed on the field data sets with sufficient data (ln-transformed) for statistical testing in Statistica 7.0 software package (StatSoft Inc., Tulsa, OK, USA), and any differences were considered significant at *p* < 0.05.

## Figures and Tables

**Table 1:** Samples of *Radix balthica* snails, infections with *Trichobilharzia* sp. “peregra”, data on the experimental setup, and daily output rates of cercariae (mean number of cercariae released per snail/day, i.e., pooled across snails and experimental days) during field and laboratory experiments. Patent infections refer to snails releasing cercariae and prepatent infections to snails that harbored sporocysts and immature cercariae.

Snail Samples	2016	2016	2017	2017	2018	2018		
	August	October	August	October	August	October		
No. of examined snails	286	347	434	577	725	689		
No. of infected snails with trematodes (overall prevalence, %)	67 (23.4)	72 (20.8)	150 (34.6)	153 (26.5)	235 (32.4)	144 (20.9)		
No. of infected snails with patent and prepatent *Trichobilharzia* (prevalence, %)	7 (2.5)	0	35 (8.1)	9 (1.6)	34 (4.7)	14 (2.0)		
No. of infected snails with patent *Trichobilharzia*	7	0	22	0	28	8		
**Experimental Setup**	**August 2016**		**August 2017**		**August 2018**		**October 2018**	
	**Field**	**Laboratory**	**Field**	**Laboratory**	**Field**	**Laboratory**	**Field**	**Laboratory**
No. of experimental days	2	1	3	3	3	3	3	3
No. of diel intervals monitored per 24 h	4 and 2 ^a^	4	5	5	5	2	5	2
No. of experimental snails, initial and [survived]	7 [7]	7 [7]	6 [2]	1 [1]	6 [6]	4 [3]	6 [6]	6 [6]
Water temperature, mean (range, °C)	8.3 (6.6–9.9)	13.8 (13.7–14.1)	13.0 (10.7–14.8)	13.4 (13.0–14.1)	14.8 (8.5–17.7)	14.2 (12.5–15.9)	6.4 (5.9–6.8)	5.7 (5.5–6.5)
Water illumination, mean (range, Lx)	4856 (0–49,600)	437 (0–538)	7250 (0–88,178)	397 (0–742)	5433 (0–74,400)	710 (0–1044)	903 (0–7578)	796 (0–2239)
**Daily Output Rates of Cercariae**								
Total no. from initial and [surviving] snails	2101	631	1668 (1385)	894	10,928	8763 (5575)	38	21
Range from initial and [surviving] snails	1–1295	14–222	0–520 (5–520)	104–537	29–2067	116–1604 (116–1291)	0–12	0–21
Mean ± SD from initial and [surviving] snails	150 ± 337	90 ± 72	128 ± 164 [231 ± 175]	298	607 ± 496	797 ± 519 [619 ± 377]	2 ± 4	1 ± 5

^a^ Different length of diel intervals during two days of experiment (see Table 2).

**Table 2:** Observed total and mean (in parentheses) number of cercariae of *Trichobilharzia* sp. “peregra” emerged from naturally infected *Radix balthica* snails during diel intervals under various field and laboratory conditions between 2016–2018. Data are pooled across snail individuals and displayed for each day of experiment separately. Cercarial emergence during the longest parts of the natural photoperiod in summer (day interval in August) and autumn (night interval in October) are divided into half and termed as Day 1, Day 2 and Night 1, Night 2.

Season/Type of Experiment	Experimental Day	Observed Number of Emerged Cercariae (Mean) during Diel Intervals				
August 2016		0:00–6:00	6:00–12:00	12:00–18:00	18:00–0:00	
Field	Day 1 (n ^a^ = 7)	1704 (243)	131 (19)	114 (16)	20 (3)	
				**12:00–0:00**		**0:00–12:00**
	Day 2 (n = 7)			114 (16)		18 (3)
		**0:00–6:00**	**6:00–12:00**	**12:00–18:00**	**18:00–0:00**	
Laboratory	Day 1 (n = 7)	518 (74)	>80 (11)	>25 (4)	>8 (1)	>
**August**		**Sunrise**	**Day 1**	**Day 2**	**Sunset**	**Night**
**2017**		**2:00–4:30**	**4:33–13:15**	**13:15–22:00**	**22:00–0:30**	**0:30–2:00**
Field	Day 1 (n = 6)	135 (23)	238 (40)	91 (15)	34 (6)	7 (1)
	Day 2 (n = 5)	244 (49)	349 (70)	151 (30)	1 (0.2)	1 (0.2)
	Day 3 (n = 2)	342 (171)	35 (18)	20 (10)	14 (7)	6 (3)
Laboratory	Day 1 (n = 1)	91	25	1	136	0
	Day 2 (n = 1)	528	5	0	4	0
	Day 3 (n = 1)	84	15	0	5	0
**August**		**Sunrise**	**Day 1**	**Day 2**	**Sunset**	**Night**
**2018**		**1:30–4:00**	**4:00–12:45**	**12:45–21:30**	**21:30–0:00**	**0:00–1:30**
Field	Day 1 (n = 6)	4032 (672)	554 (92)	9 (2)	23 (4)	1 (0.2)
	Day 2 (n = 6)	2924 (487)	511 (85)	17 (3)	0 (0)	1 (0.2)
	Day 3 (n = 6)	1642 (247)	688 (115)	121(20)	140 (23)	265 (44)
			**9:00–21:00**		**21:00–9:00**	
Laboratory	Day 1 (n = 4)		84 (21)		3868 (967)	
	Day 2 (n = 4)		9 (2)		2968 (742)	
	Day 3 (n = 3)		6 (2)		1828 (609)	
**October**		**Sunrise**	**Day**	**Sunset**	**Night 1**	**Night 2**
**2018**		**7:00–8:00**	**8:00–17:15**	**17:15–18:15**	**18:15–0:40**	**0:40–7:00**
Field	Day 1 (n = 6)	1 (0.2)	7 (1)	0 (0)	1 (0.2)	2 (0.3)
	Day 2 (n = 6)	7 (1)	6 (1)	1 (0.2)	1 (0.2)	1 (0.2)
	Day 3 (n = 6)	6 (1)	5 (1)	0 (0)	0 (0)	0 (0)
			**9:00–21:00**		**21:00–9:00**	
Laboratory	Day 1 (n = 6)		0 (0)		0 (0)	
	Day 2 (n = 6)		0 (0)		0 (0)	
	Day 3 (n = 6)		13 (2)		8 (1)	

^a^ Number of experimental snails.

**Table 3:** Results of correlation analyses testing the relationship between the number of *Trichobilharzia* sp. “peregra” cercariae emerged from naturally infected *Radix balthica* snails in field experiments and water temperature, light intensity or snail shell length. Results are displayed for each day of experiment separately and pooled across days. Statistically significant results (at α = 0.05) are indicated in bold.

Season/ Experimental Dday	No. of Snails	Temperature		Light Intensity		Snail Length	
		*r* ^a^	*p* ^b^	*r*	*p*	*r*	*p*
**August 2016**							
Day 1	6	−0.680	0.320	−0.317	0.683	0.463	0.296
Day 2	6	– ^c^	–	– ^c^	–	−0.358	0.430
Pooled data	6	– ^c^	–	– ^c^	–	0.382	0.397
**August 2017**							
Day 1	6	−0.164	0.793	0.080	0.897	0.486	0.329
Day 2	5	0.042	0.947	0.265	0.667	−0.004	0.995
Day 3	2	0.416	0.486	−0.495	0.397	– ^d^	–
Pooled data	6	0.145	0.606	−0.103	0.715	0.251	0.632
**August 2018**							
Day 1	6	−0.224	0.717	−0.012	0.985	0.329	0.525
Day 2	6	−0.276	0.653	−0.295	0.630	0.233	0.657
Day 3	6	0.713	0.176	−0.650	0.235	0.497	0.316
Pooled data	6	−0.116	0.717	−0.281	0.352	0.511	0.301
**October 2018**							
Day 1	6	−0.873	0.053	0.510	0.380	0.958	**0.003**
Day 2	6	−0.401	0.503	−0.149	0.811	0.790	0.062
Day 3	6	0.406	0.498	−0.119	0.849	0.740	0.092
Pooled data	6	−0.018	0.948	−0.080	0.776	0.844	**0.034**

^a^ Pearson’s correlation coefficient, ^b^ level of significance, ^c^ Not evaluated due to 12 h diel interval, and ^d^ Not evaluated because only two snails were available.

## Data Availability

Data supplemental to the main text are provided in Appendix A. Raw and processed data can be shared on reasonable personal request directly from the corresponding author.

## Appendix A

**Table A1:** Observed number of cercariae of *Trichobilharzia* sp. “peregra” emerged from naturally infected *Radix balthica* snails during diel intervals under various conditions in field and laboratory experiments conducted between 2016–2018. Data are shown for each snail individual and day of experiment. Cercarial emergence during the longest parts of the natural photoperiod in summer (day interval in August) and autumn (night interval in October) are divided into half and termed as Day 1, Day 2 and Night 1, Night 2. Snails that died before or during experiments are marked in grey.

Season	Snail Code				Observed Number of Emerged Cercariae during Diel Intervals											
		DAY 1					DAY 2					DAY 3				
August 2016		0:00– 6:00	6:00– 12:00	12:00– 18:00	18:00– 0:00				12:00– 00:00	00:00– 12:00		0:00– 6:00	6:00– 12:00	12:00– 18:00	18:00– 0:00	
Field	1 T-A16	238	26	12	1				14	16		– ^a^	–	–	–	
	2 T-A16	1263	4	10	18				0	1		–	–	–	–	
	3 T-A16	3	0	53	0				1	31		–	–	–	–	
	4 T-A16	16	57	4	0				0	1		–	–	–	–	
	5 T-A16	57	22	3	0				1	0		–	–	–	–	
	6 T-A16	57	22	6	1				2	57		–	–	–	–	
	7 T-A16	70	0	26	0				0	8		–	–	–	–	
Laboratory	1 T-A16	– ^a^	–	–	–				– ^a^	–		19	48	0	1	
	2 T-A16	–	–	–	–				–	–		96	18	2	1	
	3 T-A16	–	–	–	–				–	–		40	0	1	1	
	4 T-A16	–	–	–	–				–	–		208	0	14	0	
	5 T-A16	–	–	–	–				–	–		10	1	2	1	
	6 T-A16	–	–	–	–				–	–		112	9	6	2	
	7 T-A16	–	–	–	–				–	–		33	4	0	2	
**August**		**Sunrise**	**Day 1**	**Day 2**	**Sunset**	**Night**	**Sunrise**	**Day 1**	**Day 2**	**Sunset**	**Night**	**Sunrise**	**Day 1**	**Day 2**	**Sunset**	**Night**
**2017**		**2:00**– **4:30**	**4:30**– **13:15**	**13:15**– **22:00**	**22:00**– **0:30**	**0:30**– **2:00**	**2:00**– **4:30**	**4:30**– **13:15**	**13:15**– **22:00**	**22:00**– **0:30**	**0:30**– **2:00**	**2:00**– **4:30**	**4:30**– **13:15**	**13:15**– **22:00**	**22:00**– **0:30**	**0:30**– **2:00**
Field	1 T-A17	1	0	0	0	0	0	0	0	0	1	– ^b^				
	2 T-A17	4	0	1	0	0	180	24	0	1	0	288	6	2	0	2
	3 T-A17	0	2	1	5	0	7	9	3	0	0	– ^b^				
	4 T-A17	14	156	64	18	1	1	0	0	0	0	– ^b^				
	5 T-A17	116	80	25	11	6	56	316	148	0	0	54	29	18	14	4
	6 T-A17	0	0	0	0	0	– ^b^									
Laboratory	1 T-A17															
	2 T-A17	91	25	1	136	0	528	5	0	4	0	84	15	0	5	0
	3 T-A17															
	4 T-A17															
	5 T-A17	– ^c^														
	6 T-A17															
**August**		**Sunrise**	**Day 1**	**Day 2**	**Sunset**	**Night**	**Sunrise**	**Day 1**	**Day 2**	**Sunset**	**Night**	**Sunrise**	**Day 1**	**Day 2**	**Sunset**	**Night**
**2018**		**1:30**– **4:00**	**4:00**– **12:45**	**12:45**– **21:30**	**21:30**– **0:00**	**0:00**– **1:30**	**1:30**– **4:00**	**4:00**– **12:45**	**12:45**– **21:30**	**21:30**– **0:00**	**0:00**– **1:30**	**1:30**– **4:00**	**4:00**– **12:45**	**12:45**– **21:30**	**21:30**– **0:00**	**0:00**– **1:30**
Field	1 T-A18	312	14	0	0	0	380	16	0	0	0	28	1	0	0	0
	2 T-A18	1840	204	5	18	0	700	96	3	0	0	416	37	68	140	260
	3 T-A18	192	59	0	0	0	376	8	1	0	0	35	5	0	0	0
	4 T-A18	544	0	1	5	1	748	184	2	0	0	11	22	7	0	1
	5 T-A18	756	264	1	0	0	416	180	8	0	1	428	600	44	0	3
	6 T-A18	388	13	2	0	0	304	27	3	0	0	724	23	2	0	1
Laboratory			**9:00**– **21:00**		**21:00**– **9:00**			**9:00**– **21:00**		**21:00**– **9:00**			**9:00**– **21:00**		**21:00**– **9:00**	
	1 T-A18		0		684			0		284			0		352	
	2 T-A18		– ^c^													
	3 T-A18		1		372			0		116			0		652	
	4 T-A18		40		1564			4		1580			– ^b^			
	5 T-A18		43		1248			5		988			6		824	
	6 T-A18		– ^c^													
**October**		**Sunrise**	**Day**	**Sunset**	**Night 1**	**Night 2**	**Sunrise**	**Day**	**Sunset**	**Night 1**	**Night 2**	**Sunrise**	**Day**	**Sunset**	**Night 1**	**Night 2**
**2018**		**7:00**– **8:00**	**8:00**– **17:15**	**17:15**– **18:15**	**18:15**– **0:40**	**0:40**– **7:00**	**7:00**– **8:00**	**8:00**– **17:15**	**17:15**– **18:15**	**18:15**– **0:40**	**0:40**– **7:00**	**7:00**– **8:00**	**8:00**– **17:15**	**17:15**– **18:15**	**18:15**– **0:40**	**0:40**– **7:00**
Field	1 T-O18	0	0	0	0	0	0	0	0	0	0	0	0	0	0	0
	2 T-O18	0	0	0	0	0	0	0	0	0	0	0	0	0	0	0
	3 T-O18	0	0	0	0	0	0	1	0	1	1	3	0	0	0	0
	4 T-O18	0	0	0	0	0	0	0	0	0	0	0	0	0	0	0
	5 T-O18	0	2	0	0	0	0	0	1	0	0	1	0	0	0	0
	6 T-O18	1	5	0	1	2	7	5	0	0	0	2	5	0	0	0
Laboratory			**9:00**– **21:00**		**21:00**– **9:00**			**9:00**– **21:00**		**21:00**– **9:00**			**9:00**– **21:00**		**21:00**– **9:00**	
	1 T-O18		0		0			0		0			0		0	
	2 T-O18		0		0			0		0			0		0	
	3 T-O18		0		0			0		0			0		0	
	4 T-O18		0		0			0		0			0		0	
	5 T-O18		0		0			0		0			0		0	
	6 T-O18		0		0			0		0			13		8	

^a^ Experiments not performed in a given day, ^b^ Snail died during the experiment, and ^c^ Snail died before the experiment.

**Table A2:** Shell size of experimental *Radix balthica* snails infected with *Trichobilharzia* sp. “peregra” and their infection status during field and laboratory experiments on cercarial emergence conducted between 2016–2018. Patent infections refer to snails releasing cercariae and prepatent infections to snails that harbored sporocysts and immature cercariae.

Season/Year	Snail Code	Length (mm)	Width (mm)	Double Infection Identity	Double Infection Status
**August 2016**	1 T-A16	14.51	8.91	–	–
	2 T-A16	15.73	10.30	–	–
	3 T-A16	12.81	7.44	–	–
	4 T-A16	16.12	11.05	–	–
	5 T-A16	11.06	7.44	–	–
	6 T-A16	11.76	7.70	–	–
	7 T-A16	15.33	9.76	*Plagiorchis* sp.	patent
**August 2017**	1 T-A17	8.69	5.16	–	–
	2 T-A17	10.45	8.84	–	–
	3 T-A17	15.36	12.44	–	–
	4 T-A17	14.76	12.73	*Plagiorchis* sp.	prepatent
	5 T-A17	12.81	10.93	–	–
	6 T-A17	12.77	10.89	*Apatemon* sp.	prepatent
**August 2018**	1 T-A18	10.57	6.15	–	–
	2 T-A18	12.27	7.98	–	–
	3 T-A18	9.57	5.87	–	–
	4 T-A18	12.80	7.78	–	–
	5 T-A18	11.26	6.23	–	–
	6 T-A18	13.92	8.06	–	–
**October 2018**	1 T-O18	12.63	8.25	*Plagiorchis* sp.	prepatent
	2 T-O18	9.17	5.79	–	–
	3 T-O18	11.11	6.85	–	–
	4 T-O18	11.17	6.54	*Plagiorchis* sp.	prepatent
	5 T-O18	15.11	8.60	*Cotylurus* sp.	prepatent
	6 T-O18	19.96	11.14	*Diplostomum* sp.	prepatent

**Table A3:** Mean water temperature (°C) and light intensity (Lx) during diel intervals under various field and laboratory conditions between 2016–2018. Data are displayed for each day of experiment separately. Cercarial emergence during the longest parts of the natural photoperiod in summer (day interval in August) and autumn (night interval in October) are divided into half and termed as Day 1, Day 2 and Night 1, Night 2. Data marked in yellow represent additional data illustrating/simulating natural thermo- and photo-conditions in the field or a climatic chamber (note that cercarial emergence was not investigated according to these temperature and light conditions; see Materials and Methods for experimental conditions).

Season/Year	Type of Experiment	Experimental Day		Temperature and Light Intensity during Diel Intervals				
August 2016				0:00–6:00	6:00–12:00	12:00–18:00	18:00–0:00	
	Field	Day 1	°C	7.8	8.5	9.2	8.4	
			Lx	415	6962	13821	1216	
						**12:00–0:00**		**0:00–12:00**
		Day 2	°C			8.8		7.3
			Lx			5803		2311
				**Sunrise** **2:00–4:30**	**Day 1** **4:33–13:15**	**Day 2** **13:15–22:00**	**Sunset** **22:00–0:30**	**Night** **0:30–2:00**
	Field	Day 1	°C	7.8	8.4	9.0	7.8	7.7
			Lx	108	6321	9107	9	0
		Day 2	°C	6.8	7.4	9.1	7.7	7.2
			Lx	120	3448	6823	7	0
				**0:00–6:00**	**6:00–12:00**	**12:00–18:00**	**18:00–0:00**	
	Laboratory	Day 1	°C	13.7	13.8	13.8	13.7	
			Lx	372	538	505	311	
**August 2017**				**Sunrise** **2:00–4:30**	**Day 1** **4:33–13:15**	**Day 2** **13:15–22:00**	**Sunset** **22:00–0:30**	**Night** **0:30–2:00**
	Field	Day 1	°C	12.8	13.1	12.9	13.0	12.9
			Lx	143	7659	5871	37	0
		Day 2	°C	12.6	13.1	13.8	12.8	12.7
			Lx	71	8370	13145	16	0
		Day 3	°C	13.0	12.7	12.5	13.2	13.1
			Lx	153	11024	13647	14	0
	Laboratory	Day 1	°C	13.4	13.6	13.7	13.4	13.3
			Lx	31	535	533	77	0
		Day 2	°C	13.1	13.2	13.2	13.3	13.2
			Lx	60	571	511	55	0
		Day 3	°C	13.1	13.3	13.3	13.1	13.1
			Lx	27	553	491	27	0
**August 2018**				**Sunrise** **1:30–4:00**	**Day 1** **4:00–12:45**	**Day 2** **12:45–21:30**	**Sunset** **21:30–0:00**	**Night** **0:00–1:30**
	Field	Day 1	°C	15.4	15.7	16.3	15.9	14.9
			Lx	30	13949	5428	146	0
		Day 2	°C	15.9	11.5	16.9	16.3	16.0
			Lx	11	2960	12926	17	0
		Day 3	°C	14.4	14.5	13.1	14.1	14.1
			Lx	12	4787	4591	63	0
					**9:00–21:00**		**21:00–9:00**	
	Laboratory	Day 1	°C		14.2		14.1	
			Lx		965		413	
		Day 2	°C		14.2		14.1	
			Lx		978		466	
		Day 3	°C		14.3		14.1	
			Lx		965		471	
				**Sunrise** **1:30–4:00**	**Day 1** **4:00–12:45**	**Day 2** **12:45–21:30**	**Sunset** **21:30–0:00**	**Night** **0:00–1:30**
	Laboratory	Day 1	°C	14.1	14.2	14.2	14.1	14.1
			Lx	10	926	976	89	0
		Day 2	°C	14.0	14.1	14.2	14.1	14.1
			Lx	10	965	983	115	0
		Day 3	°C	14.1	14.2	14.2	14.1	14.0
			Lx	9	954	967	150	0
**October 2018**				**Sunrise**	**Day**	**Sunset**	**Night 1**	**Night 2**
				**7:00–8:00**	**8:00–17:15**	**17:15–18:15**	**18:15–0:40**	**0:40–7:00**
	Field	Day 1	°C	6.3	6.4	6.6	6.5	6.4
			Lx	236	2198	6	0	0
		Day 2	°C	6.3	6.5	6.6	6.5	6.3
			Lx	109	1786	70	0	0
		Day 3	°C	6.5	6.6	6.3	6.2	6.5
			Lx	43	2929	39	0	0
					**9:00–21:00**		**21:00–9:00**	
	Laboratory	Day 1	°C		5.8		5.7	
			Lx		1367		161	
		Day 2	°C		5.7		5.7	
			Lx		1,456		183	
		Day 3	°C		5.8		5.7	
			Lx		1429		186	
				**Sunrise**	**Day**	**Sunset**	**Night 1**	**Night 2**
				**7:00–8:00**	**8:00–17:15**	**17:15–18:15**	**18:15–0:40**	**0:40–7:00**
	Laboratory	Day 1	°C	6.0	5.8	5.6	5.6	5.7
			Lx	103	1954	102	0	0
		Day 2	°C	6.0	5.7	5.6	5.7	5.7
			Lx	103	2095	176	0	0
		Day 3	°C	6.1	5.8	5.6	5.7	5.7
			Lx	108	2075	35	0	0

## References

[1] Colley D.G., Bustinduy A.L., Secor W.E., King C.H. (2014). Human schistosomiasis. Lancet.

[2] Jamieson B.G.M. (2017). Schistosoma: Biology, Pathology and Control.

[3] Brant S.V., Loker E.S. (2009). Molecular systematics of the avian schistosome genus *Trichobilharzia* (Trematoda: Schistosomatidae) in North America. J. Parasitol..

[4] Soldánová M., Selbach C., Kalbe M., Kostadinova A., Sures B. (2013). Swimmer’s itch: Etiology, impact, and risk factors in Europe. Trends Parasitol..

[5] Horák P., Mikeš L., Lichtenbergová L., Skála V., Soldánová M., Brant S.V. (2015). Avian schistosomes and outbreaks of cercarial dermatitis. Clin. Microbiol. Rev..

[6] Galaktionov K.V., Dobrovolskij A. (2003). The Biology and Evolution of Trematodes: An Essay on the Biology, Morphology, Life Cycles, Transmissions, and Evolution of Digenetic Trematodes.

[7] Pandian T.J. (2020). Reproduction and Development in Platyhelminthes.

[8] Combes C., Fournier A., Moné H., Théron A. (1994). Behaviours in trematode cercariae that enhance parasite transmission: Patterns and processes. Parasitology.

[9] Joosse J., van Elk R. (1986). *Trichobilharzia ocellata*: Physiological characterization of giant growth, glycogen depletion, and absence of reproductive activity in the intermediate snail host, *Lymnaea stagnalis*. Exp. Parasitol..

[10] Gérard C., Théron A. (1997). Age/size- and time-specific effects of *Schistosoma mansoni* on energy allocation patterns of its snail host *Biomphalaria glabrata*. Oecologia.

[11] Żbikowska E., Marszewska A. (2018). Thermal preferences of bird schistosome snail hosts increase the risk of swimmer’s itch. J. Therm. Biol..

[12] Skála V., Walker A.J., Horák P. (2020). Snail defence responses to parasite infection: The *Lymnaea stagnalis*-*Trichobilharzia szidati* model. Dev. Comp. Immunol..

[13] Loker E.S. (1983). A comparative study of the life-histories of mammalian schistosomes. Parasitology.

[14] Haas W. (1994). Physiological analyses of host-finding behaviour in trematode cercariae: Adaptations for transmission success. Parasitology.

[15] Saap K.K., Loker E.S. (2000). Mechanisms underlying digenean-snail specificity: Role of miracidial attachment and host plasma factors. J. Parasitol..

[16] Haas W. (2003). Parasitic worm: Strategies of host finding, recognition and invasion. Zoology.

[17] Combes C., Bartoli P., Théron A., Lewis E.E., Campbell J.F., Sukhdeo M.V.K. (2002). Trematode transmission strategies. The Behavioural Ecology of Parasites.

[18] Morley N.J. (2012). Cercariae (Platyhelminthes: Trematoda) as neglected components of zooplankton communities in freshwater habitats. Hydrobiologia.

[19] Esch G.W., Curtis L.A., Barger M.A. (2001). A perspective on the ecology of trematode communities in snails. Parasitology.

[20] Théron A. (2015). Chronobiology of trematode cercarial emergence: From data recovery to epidemiological, ecological and evolutionary implications. Adv. Parasitol..

[21] Mouahid G., Idris M.A., Verneau O., Théron A., Shaban M.M., Moné H. (2012). A new chronotype of *Schistosoma mansoni*: Adaptive significance. Trop. Med. Int. Health.

[22] Mouahid G., Mintsa Nguema R., Al Mashikhi K.M., Al Yafae S.A., Idris M.A., Moné H. (2019). Host-parasite life-histories of the diurnal vs. nocturnal chronotypes of *Schistosoma mansoni*: Adaptive significance. Trop. Med. Int. Health.

[23] Wang S.R., Zhu Y.J., Ge Q.P., Yang M.J., Huang J.L., Huang W.Q., Zhuge H.X., Lu D.B. (2015). Effect of photoperiod change on chronobiology of cercarial emergence of *Schistosoma japonicum* derived from hilly and marshy regions of China. Exp. Parasitol..

[24] Cort W.W. (1950). Studies on schistosome dermatitis XI. Status of knowledge after more than 20 years. Am. J. Hyg..

[25] Neuhaus W. (1952). Biologie und Entwicklung von *Trichobilharzia szidati* n. sp. (Trematoda Schistosomatidae), einem Erreger von Dermatitis bei Menschen. Z. Parasitenkd..

[26] Chernogorenko M.I., Boryak Y.V. (1973). The biology of cercariae of *Trichobilharzia ocellata* La Val., 1854. Gidrobiol. Zhurnal.

[27] Anderson P.A., Nowosielski J.W., Croll N.A. (1976). The emergence of cercariae of *Trichobilharzia ocellata* and its relationship to the activity of its snail host *Lymnaea stagnalis*. Can. J. Zool..

[28] Appleton C.C., Lethbridge R.C. (1979). Schistosome dermatitis in the Swan Estuary, Western Australia. Med. J. Aust..

[29] Sluiters J.E., Brussaard-Wust C.M., Meuleman E.A. (1980). The relationship between miracidial dose, production of cercariae, and reproductive activity of the host in the combination *Trichobilharzia ocellata* and *Lymnaea stagnalis*. Z. Parasitenkd..

[30] Leighton B.J., Zervos S., Webster J.M. (2000). Ecological factors in schistosome transmission, and an environmentally benign method for controlling snails in a recreational lake with a record of schistosome dermatitis. Parasitol. Int..

[31] Soldánová M., Selbach C., Sures B. (2016). The early worm catches the bird? Productivity and patterns of *Trichobilharzia szidati* cercarial emission from *Lymnaea stagnalis*. PLoS ONE.

[32] Rudko S.P., Reimink R.L., Froelich K., Gordy M.A., Blankespoor C.L., Hanington P.C. (2018). Use of qPCR-based cercariometry to assess swimmer’s itch in recreational lakes. Ecohealth.

[33] Al-Jubury A., Kania P., Bygum A., Buchmann K. (2020). Temperature and light effects on *Trichobilharzia szidati* cercariae with implications for a risk analysis. Acta. Vet. Scand..

[34] Oyarzún-Ruiz P., Thomas P., Santodomingo A., Collado G., Muñoz P., Moreno L. (2022). Morphological, behavioral, and molecular characterization of avian schistosomes (Digenea: Schistosomatidae) in the native snail *Chilina dombeyana* (Chilinidae) from Southern Chile. Pathogens.

[35] Rojo-Vazquez F.A., Simon-Martin F. (1985). Algunos aspectos de la biología de las cercarias de *Trichobilharzia* sp. del Rio Canedo (Provinciade Salamanca, Espana). Rev. Iber. Parasitol..

[36] Rind S. (1991). Three ocellate schistosome cercariae (Trematoda: Schistosomatidae) in *Gyraulus corinna*, with reference to *Cercaria longicauda* MacFarlane, 1944 in *Lymnaea tomentosa*. N. Z. J. Zool..

[37] Shakarbayev U.A., Akramova F.D., Norkobilov B.T., Azimov D.A. (2020). Cercariae of trematodes of mollusks (Gastropoda, Pulmonates) in reservoirs of Uzbekistan. Pharma Innov. J..

[38] Shostak A.W., Esch G.W. (1990). Photocycle-dependent emergence by cercariae of *Halipegus occidualis* from *Helisoma anceps*, with special reference to cercarial emergence patterns as adaptations for transmission. J. Parasitol..

[39] Poulin R. (2006). Global warming and temperature-mediated increases in cercarial emergence in trematode parasites. Parasitology.

[40] Morley N.J., Lewis J.W. (2013). Thermodynamics of cercarial development and emergence in trematodes. Parasitology.

[41] Selbach C., Poulin R. (2020). Some like it hotter: Trematode transmission under changing temperature conditions. Oecologia.

[42] Wolmarans C.T., de Kock K.N., Strauss H.D., Bornman M. (2002). Daily emergence of *Schistosoma mansoni* and *S*. *haematobium* cercariae from naturally infected snails under field conditions. J. Helminthol..

[43] Lu D.B., Wang T.P., Rudge J.W., Donnelly C.A., Fang G.R., Webster J.P. (2009). Evolution in a multi-host parasite: Chronobiological circadian rhythm and population genetics of *Schistosoma japonicum* cercariae indicates contrasting definitive host reservoirs by habitat. Int. J. Parasitol..

[44] Skírnisson K., Aldhoun J.A., Kolářová L. (2009). A review on swimmer’s itch and the occurrence of bird schistosomes in Iceland. J. Helminthol..

[45] Nikolaev K.E., Levakin I.A., Galaktionov K.V. (2021). A month for the mission: Using a sentinel approach to determine the transmission window of digenean cercariae in the subarctic White Sea. J. Helminthol..

[46] Galaktionov K.V. (2017). Patterns and processes influencing helminth parasites of Arctic coastal communities during climate change. J. Helminthol..

[47] Brassard P., Curtis M.A., Rau M.E. (1982). Seasonality of *Diplostomum spathaceum* (Trematoda, Strigeidae) transmission to brook trout (*Salvelinus fontinalis*) in northern Quebec, Canada. Can. J. Zool..

[48] Prokofiev V.V., Galaktionov K.V., Levakin I.A., Nikolaev K.E. (2020). Light or temperature? What regulates the emergency of trematode cercariae from the molluscan hosts and how it is done. Parazitologiya.

[49] Larsen A.H., Bresciani J., Buchmann K. (2004). Increasing frequency of cercarial dermatitis at higher latitudes. Acta Parasitol..

[50] Aldhoun J.A., Faltýnková A., Karvonen A., Horák P. (2009). Schistosomes in the North: A unique finding from a prosobranch snail using molecular tools. Parasitol. Int..

[51] Soleng A., Mehl R. (2011). Geographical distribution of cercarial dermatitis in Norway. J. Helminthol..

[52] Gordy M.A., Cobb T.P., Hanington P.C. (2018). Swimmer’s itch in Canada: A look at the past and survey of the present to plan for the future. Environ. Health.

[53] Gordy M.A., Hanington P.C. (2019). A fine-scale phylogenetic assessment of digenean trematodes in central Alberta reveals we have yet to uncover their total diversity. Ecol. Evol..

[54] Soldánová M., Georgieva S., Roháčová J., Knudsen R., Kuhn J.A., Henriksen E.H., Siwertsson A., Shaw J.C., Kuris A.M., Amundsen P.-A. (2017). Molecular analyses reveal high species diversity of trematodes in a sub-Arctic lake. Int. J. Parasitol..

[55] Born-Torrijos A., Paterson R.A., van Beest G.S., Schwelm J., Vyhlídalová T., Henriksen E.H., Knudsen R., Kristoffersen R., Amundsen P.-A., Soldánová M. (2020). Temperature does not influence functional response of amphipods consuming different trematode prey. Parasitol. Res..

[56] Born-Torrijos A., Paterson R.A., van Beest G.S., Vyhlídalová T., Henriksen E.H., Knudsen R., Kristoffersen R., Amundsen P.-A., Soldánová M. (2021). Cercarial behaviour alters the consumer functional response of three-spined sticklebacks. J. Anim. Ecol..

[57] Born-Torrijos A., van Beest G.S., Vyhlídalová T., Knudsen R., Kristoffersen R., Amundsen P., Thieltges D.W., Soldánová M. (2022). Taxa-specific activity loss and mortality patterns in freshwater trematode cercariae under subarctic conditions. Parasitology.

[58] Soldánová M., Kundid P., Scholz T., Kristoffersen R., Knudsen R. (2022). Somatic dimorphism in cercariae of a bird schistosome. Pathogens.

[59] Marcogliese D.J. (2001). Implications of climate change for parasitism of animals in the aquatic environment. Can. J. Zool..

[60] Kutz S.J., Jenkins E.J., Veitch A.M., Ducrocq J., Polley L., Elkin B., Lair S. (2009). The Arctic as a model for anticipating, preventing, and mitigating climate change impacts on host-parasite interactions. Vet. Parasitol..

[61] Mas-Coma S., Valero A.V., Bargues M.D. (2009). Climate change effects on trematodiases, with emphasis on zoonotic fascioliasis and schistosomiasis. Vet. Parasitol..

[62] Hoberg E.P., Cook J.A., Agosta S.J., Boeger W., Galbreath K.E., Laaksonen S., Kutz S.J., Brooks D.R. (2017). Arctic systems in the Quaternary: Ecological collision, faunal mosaics and the consequences of a wobbling climate. J. Helminthol..

[63] Lashaki E.K., Teshnizi S.H., Gholami S., Fakhar M., Brant S.V., Dodangeh S. (2020). Global prevalence status of avian schistosomes: A systematic review with meta-analysis. Parasite Epidemiol. Control.

[64] Prokofiev V.V., Galaktionov K.V., Levakin I.A. (2016). Patterns of parasite transmission in polar seas: Daily rhythms of cercarial emergence from intertidal snails. J. Sea Res..

[65] Vyhlídalová T., Soldánová M. (2020). Species-specific patterns in cercarial emergence of *Diplostomum* spp. from snails *Radix lagotis*. Int. J. Parasitol..

[66] Anderson R.M., May R.M. (1979). Prevalence of schistosome infections within molluscan populations: Observed patterns and theoretical predictions. Parasitology.

[67] N’Goran E., Brémond P., Sellin E., Sellin B., Théron A. (1997). Intraspecific diversity of *Schistosoma haematobium* in west Africa: Chronobiology of cercarial emergence. Acta Trop..

[68] Noda S., Sato K., Katsumata T., Nojima H., Muhoho N.D. (1986). The influence of shadowing on emergence of *Schistosoma haematobium* during day time. Jpn. J. Parasitol..

[69] Arp C.D., Jones B.M., Whitman M., Larsen A., Urban F.E. (2010). Lake temperature and ice cover regimes in the Alaskan Subarctic and Arctic: Integrated monitoring, remote sensing, and modeling. J. Am. Water Resour. Assoc..

[70] Thieltges D.W., Rick J. (2006). Effect of temperature on emergence, survival and infectivity of cercariae of the marine trematode *Renicola roscovita* (Digenea: Renicolidae). Dis. Aquat. Org..

[71] Thieltges D.W., Jensen K.T., Poulin R. (2008). The role of biotic factors in the transmission of free-living endohelminth stages. Parasitology.

[72] Johnson P.T.J., Dobson A., Lafferty K.D., Marcogliese D.J., Memmott J., Orlofske S.A., Poulin R., Thieltges D.W. (2010). When parasites become prey: Ecological and epidemiological significance of eating parasites. Trends Ecol. Evol..

[73] Koprivnikar J. (2022). The enemy of my enemy is my friend: Consumption of parasite infectious stages benefits hosts and predators depending on transmission mode. J. Anim. Ecol..

[74] Morley N.J., Adam M.E., Lewis J.W. (2010). The effects of host size and temperature on the emergence of *Echinoparyphium recurvatum* cercariae from *Lymnaea peregra* under natural light conditions. J. Helminthol..

[75] Graham L.A. (2003). Effect of snail size on the prevalence and intensity of avian schistosome infection: Relating laboratory to field studies. J. Parasitol..

[76] Lim H.K., Heyneman D. (1972). Intramolluscan inter-trematode antagonism: A review of factors influencing the host-parasite system and its possible role in biological control. Adv. Parasitol..

[77] McCarthy A.M. (1999). Photoperiodic cercarial emergence patterns of the digeneans *Echinoparyphium recurvatum* and *Plagiorchis* sp. from a mixed infection in *Lymnaea peregra*. J. Helminthol..

[78] Soldánová M., Kuris A.M., Scholz T., Lafferty K.D. (2012). The role of spatial and temporal heterogeneity and competition in structuring trematode communities in the great pond snail, *Lymnaea stagnalis* (L.). J. Parasitol..

[79] Massoud J. (1974). The effect of variation in miracidial exposure dose on laboratory infections of *Ornithobilharzia turkestanicum* in *Lymnaea gedrosiana*. J. Helminthol..

[80] Seppälä O., Liljeroos K., Karvonen A., Jokela J. (2008). Host condition as a constraint for parasite reproduction. Oikos.

[81] Berkhout B.W., Lloyd M.M., Poulin R., Studer A. (2014). Variation among genotypes in response to increasing temperature in a marine parasite: Evolutionary potential in the face of global warming?. Int. J. Parasitol..

[82] Klemetsen A., Knudsen R. (2013). Diversity and abundance of water birds in a subarctic lake during three decades. Fauna Norv..

[83] Sayler R.D., Afton A.D. (1981). Ecological aspects of common goldeneyes *Bucephala clangula* wintering on the upper Mississippi River. Ornis Scand..

[84] Danell K., Sjöberg K. (1982). Seasonal and diel changes in the feeding behaviour of some dabbling duck species on a breeding lake in northern Sweden. Ornis Scand..

[85] Amundsen P.-A., Primicerio R., Smalås A., Henriksen E.H., Knudsen R., Kristoffersen R., Klemetsen A. (2019). Long-term ecological studies in northern lakes - challenges, experiences, and accomplishments. Limnol. Oceanogr..

[86] Jouet D., Skírnisson K., Kolářová L., Ferté H. (2010). Molecular diversity of *Trichobilharzia franki* in two intermediate hosts (*Radix auricularia* and *Radix peregra*): A complex of species. Infect. Genet. Evol..

[87] WMO (World Meteorological Organization) (2018). 2018 Annual Report: WMO for the the Twenty-First Century.

